# Monitoring of Sitting Postures With Sensor Networks in Controlled and Free-living Environments: Systematic Review

**DOI:** 10.2196/21105

**Published:** 2021-03-01

**Authors:** Arpita Mallikarjuna Kappattanavar, Nico Steckhan, Jan Philipp Sachs, Harry Freitas da Cruz, Erwin Böttinger, Bert Arnrich

**Affiliations:** 1 Hasso-Plattner-Institut University of Potsdam Potsdam Germany; 2 Hasso Plattner Institute for Digital Health at Mount Sinai Icahn School of Medicine at Mount Sinai New York, NY United States

**Keywords:** classification, algorithms, sitting position, spine, technology, machine learning, back pain, movement, extremities

## Abstract

**Background:**

A majority of employees in the industrial world spend most of their working time in a seated position. Monitoring sitting postures can provide insights into the underlying causes of occupational discomforts such as low back pain.

**Objective:**

This study focuses on the technologies and algorithms used to classify sitting postures on a chair with respect to spine and limb movements, using sensors and wearables such as inertial measurement units, pressure or piezoresistive sensors, accelerometers or gyroscopes, combined with machine learning approaches.

**Methods:**

A total of three electronic literature databases were surveyed to identify studies classifying sitting postures in adults. Quality appraisal was performed to extract critical details and assess biases in the shortlisted papers.

**Results:**

A total of 14 papers were shortlisted from 952 papers obtained after a systematic search. The majority of the studies used pressure sensors to measure sitting postures, whereas neural networks were the most frequently used approaches for classification tasks in this context. Only 2 studies were performed in a free-living environment. Most studies presented ethical and methodological shortcomings. Moreover, the findings indicate that the strategic placement of sensors can lead to better performance and lower costs.

**Conclusions:**

The included studies differed in various aspects of design and analysis. The majority of studies were rated as medium quality according to our assessment. Our study suggests that future work for posture classification can benefit from using inertial measurement unit sensors, since they make it possible to differentiate among spine movements and similar postures, considering transitional movements between postures, and using three-dimensional cameras to annotate the data for ground truth. Finally, comparing such studies is challenging, as there are no standard definitions of sitting postures that could be used for classification. In addition, this study identifies five basic sitting postures along with different combinations of limb and spine movements to help guide future research efforts.

## Introduction

### Background

The proportion of people sitting for long hours during work and daily life has increased in recent years. Approximately 75% of employees in call centers, software companies, and other industrial jobs spend an average of 90% of their workday sitting on a chair [1,2]. Many individuals who sit for long hours in the same posture, or *bad* posture, experience musculoskeletal discomfort and pain at the ischiocrural muscle region [3]. Prolonged sitting behavior and spine-straining sitting postures have been reported to act as negative factors, increasing the probability of developing low back pain (LBP) [1,2,4-6].

LBP has been identified as a significant cause of sick leaves and disability, leading to impairment in daily and occupational activities, reflecting a significant economic burden on the society [7-9]. The majority (90%) of LBP cases are nonspecific [8,10-12]. By definition, nonspecific LBP cases have an unknown origin, where mechanical factors and multifactorial etiology are suspected. There is still a gap in understanding whether mechanical factors are associated with nonspecific LBP, as it has not been verified in research studies [8]. Continuous monitoring of spine movements and daily activities would help understand the link between the various mechanical and psychosocial factors leading to LBP and differentiate them [12].

To implement appropriate intervention and prevention programs for LBP, especially within an office environment, identifying risk factors such as stress at work and sitting postures is of high importance according to the Bulletin of the World Health Organization (WHO) [9] and from the studies conducted by Bontrup et al [1] and Søndergaard et al [3]. Therefore, this systematic literature review focusses on the classification of sitting postures.

In traditional methods, sitting postures were analyzed by observing the seated subjects and self-reported answers to questionnaires [13]. However, these methods are biased and subjective, and vary for each doctor and patient. Therefore, the data were unreliable. With advancements in micro-electro-mechanical systems and nano-electro-mechanical systems, different types of miniaturized sensor technologies are readily available in the market. They can assess and classify sitting postures more objectively and accurately. In the last decade, studies have used miniaturized pressure sensors made from air bladders, piezoelectric materials, fibers coated with yarn materials, force sensors, and force-sensing resistors in the form of cushions, sensor array sheets, and mats or just as individual sensors to provide the necessary signals to classify sitting postures [1,13-24]. Such classifications should also preferably include limb movements, as these are suspected to be associated with musculoskeletal discomfort and pain [25-27].

### Objective

This study has been conducted to understand the state-of-the-art technologies for classifying sitting postures on a chair along with limb and spine movements. To achieve this goal, we (1) use a systematic database search approach using the Population or Problem, Intervention or Exposure, Comparison, and Outcome (PICO) scheme; (2) carry out a quality appraisal of the included papers; (3) summarize the algorithms and the number of postures classified; (4) investigate the study design and the type of environment in which these studies were conducted; and (5) identify the challenges in classifying sitting postures and critically assess the technological solutions employed.

**Figure 1:**
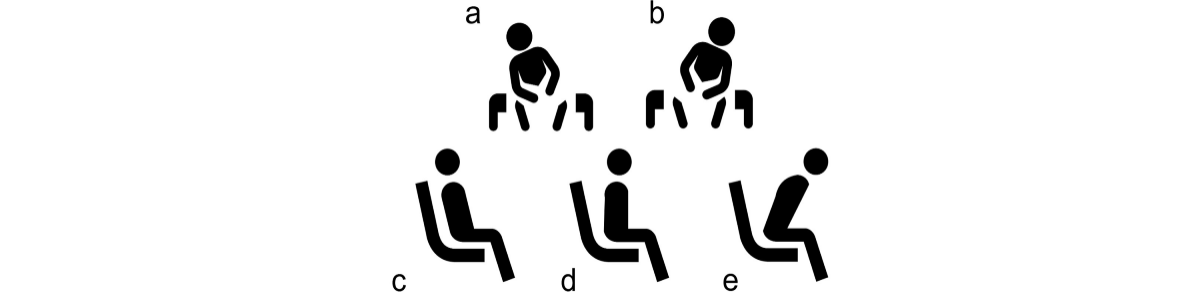
The 5 most common sitting postures: (a) lean right, (b) lean left, (c) lean backward, (d) upright sitting, and (e) lean forward.

The majority of the studies in this review used pressure sensors to measure sitting postures, whereas neural networks (NNs) were the most frequently used approaches for classification tasks in this context. In total, 5 main postures as shown in Figure 1, were presented in all studies along with different combinations of limb and spine movements.

The organization of this paper is as follows: In the *Methods* section, we present the search approach, the inclusion and exclusion criteria applied to shortlist the papers, checklists for bias assessment, and data extracted from the papers. In *Results* section, we present the summary of all the shortlisted articles and outline the details of the extracted data. In the *Discussion* section, we investigate and discuss the findings, and in the *Conclusions* section, we provide recommendations and an outlook on future work. Finally, in the *Limitations* section, we discuss the limitations of this study.

## Methods

### Search Approach

A systematic search was conducted on PubMed, IEEE Xplore, and Web of Science databases until June 2019. The literature search strategy framework in systematic reviews is typically based on the PICO scheme [28]. The search in this study was partially based on PICO. In the keywords’ formation, we included *sitting* for population or problem, the *tools or technology* was mentioned for intervention, and *algorithms* were mentioned for the outcome. As we did not have a comparison, it was excluded from PICO. The search focused on papers with the main terms and specific terms, as indicated in Table 1. Search engines’ specific terms varied slightly (Multimedia Appendix 1). Additional papers were identified by manually searching and screening the reference lists of other papers to identify papers that have been overlooked by the electronic search. Retrieved papers were imported to Mendeley Desktop.

**Table 1:** Terms for literature database search. The specific terms of the 3 main categories have been joined by an AND condition.

Main terms	Specific search terms
Sitting	(sitting OR seating OR seated) AND (posture^*^ OR position OR behaviour)
Sensor	sensor* OR “inertial measurement unit” OR IMU OR wearable OR pressure OR piezoresistive OR accelerometer OR gyroscope
Algorithm	“machine learning” OR “neural network*” OR algorithm* OR ^*^supervised OR classif* OR detection OR identification OR recognition

### Study Selection Method

The search terms were assessed by two authors independently and iteratively and then finalized. The final search terms were used to obtain papers from the aforementioned databases. The titles and abstracts of the obtained papers were carefully read and analyzed before shortlisting them based on the inclusion and exclusion criteria.

Papers that met the following criteria have been included:

1. More than 3 sitting postures were classified
2. Journal or conference papers were published in English language
3. The involved population was sitting on a chair
4. The study involved adult population (older than 18 years)

Papers have been excluded based on the following criteria, if:

1. Limb movements while sitting were not considered. Studies have revealed that leg movements affect musculoskeletal discomfort and pain [25-27].
2. The involved population was sitting in a wheelchair or driving a vehicle. The postures for a wheelchair subject were less dependent on the leg movements. Moreover, driving postures differ from those of sitting postures in occupational settings.
3. The methodology and classification accuracy of each posture was not mentioned or reported, as the classification accuracy provided the proof of the methodology for replication.
4. Duplicates were avoided if the same author mentioned the same methodology in a journal and a conference article, and then the conference article was excluded.
5. The same methodology is mentioned in 2 papers by the same authors with little variation. A paper that provided a higher level of details was included in this study.
6. A paper is not related to sitting postures.
7. Sensors were implanted inside the body, as our study focused on noninvasive methods.

Differences in the inclusion of specific papers were resolved by consulting with other authors of this study.

### Study Quality Assessment

Quality appraisal checklists were developed to extract key details and identify the risk of bias in each study. This checklist was prepared based on consultation with other authors and using the studies by Papi et al [12] and Hagströmer et al [29] as a reference to include relevant points. The prepared checklist has questions related to 3 categories, that is, study description, study design, and robustness. Table 2 presents the study quality assessment checklist questionnaires based on the three categories to assess the risk of bias.

The customized checklist is provided in Table 3. The table has been further numbered as 0, 1, or 2 for each selected paper to rate it as no detail, limited detail, and good detail, respectively. The total score is based on the sum of those checkpoint scores (0-26). These papers were rated as low (low<10), moderate (10<moderate<18), or high (19<high<26) quality based on the total score of the paper. The score for each paper was based on the discussion with other authors.

### Data Extraction

This study was conducted to investigate the technology and algorithms used to classify the sitting postures in different settings. Therefore, we extracted details concerning the technology, study design, classification algorithm, and algorithm performance from the shortlisted papers, as presented in Textbox 1. In addition to quality appraisal, these items will guide the remainder of this paper.

**Table 2:** The checklist questions to assess the risk of bias.

Categories	Checklist questions
Study description	Q1. Are the research objectives or aims stated? Q2. Is the study clearly described? Q3. Were the main findings of the study stated? Q4. Are the limitations of the study clearly described?
Study design	Q5. Are appropriate subject information and anthropometric details provided? Q6. Were the number of subjects studied justified? Q7. Was prominence of leg crossing considered? Q8. Were the eligibility criteria mentioned? Q9. Were there ethics committee approval and written consent mentioned in the papers? Q10. Was the justification for the sensor setup and location given?
Robustness	Q11. Were measures of reliability or accuracy of the algorithm reported? Q12. Were the classifications cross-validated? Q13. Is the system robust in the wild (controlled or free-living environments)?

**Table 3:** Quality bias assessment table to rate the quality of the shortlisted papers based on the three question categories described previously. Q represents the checklist question number.

Study	Study description					Study design							Robustness				Total		Quality
	Q1	Q2	Q3	Q4	Q5		Q6	Q7	Q8	Q9	Q10	Q11		Q12	Q13				
Ma et al [30]	2^a^	1^b^	1	2	0^c^		0	1	1	0	0	1		0	1	10		Medium	
Zemp et al [14]	2	2	2	2	2		2	1	1	2	0	1		1	1	19		High	
Xu et al [13]	2	1	2	1	1		0	1	0	0	1	2		0	1	12		Medium	
Martins et al [15]	2	1	2	2	2		0	2	0	0	2	2		1	1	17		Medium	
Zemp et al [16]	2	2	2	2	2		0	1	0	2	0	2		1	2	18		Medium	
Kamiya et al [17]	2	2	2	0	1		0	2	0	0	0	2		1	1	13		Medium	
Liu et al [18]	2	2	2	0	0		0	1	0	0	0	1		2	1	11		Medium	
Pereira et al [19]	2	2	1	0	2		0	2	0	0	2	2		0	1	14		Medium	
Zhu et al [20]	2	1	2	0	1		0	2	0	0	1	1		0	1	11		Medium	
Bontrup et al [1]	2	1	2	2	2		0	1	1	2	0	2		1	2	18		Medium	
Mutlu et al [21]	2	1	2	2	1		0	2	0	0	2	2		1	1	16		Medium	
Huang et al [22]	2	1	2	1	0		0	1	0	0	0	2		0	1	10		Medium	
Wang et al [23]	2	2	2	1	2		0	1	0	0	1	2		0	1	14		Medium	
Noh et al [24]	2	0	2	0	0		0	0	0	0	0	2		0	1	7		Low	

^a^2: good detail.

^b^1: limited detail.

^c^0: no detail.

**Summary of the data extracted from each of the shortlisted papers.**

Technology

- Sensor type
- The number of sensors
- Sensor location

Study design

- The environment in which these studies were performed
- The number of subjects recruited
- Study protocol

Classification algorithm

- Algorithms used
- The type of features extracted
- Number of postures classified

Algorithm performance

- Performance metrics
- Evaluation setup

## Results

### Shortlisted Papers

**Figure 2:**
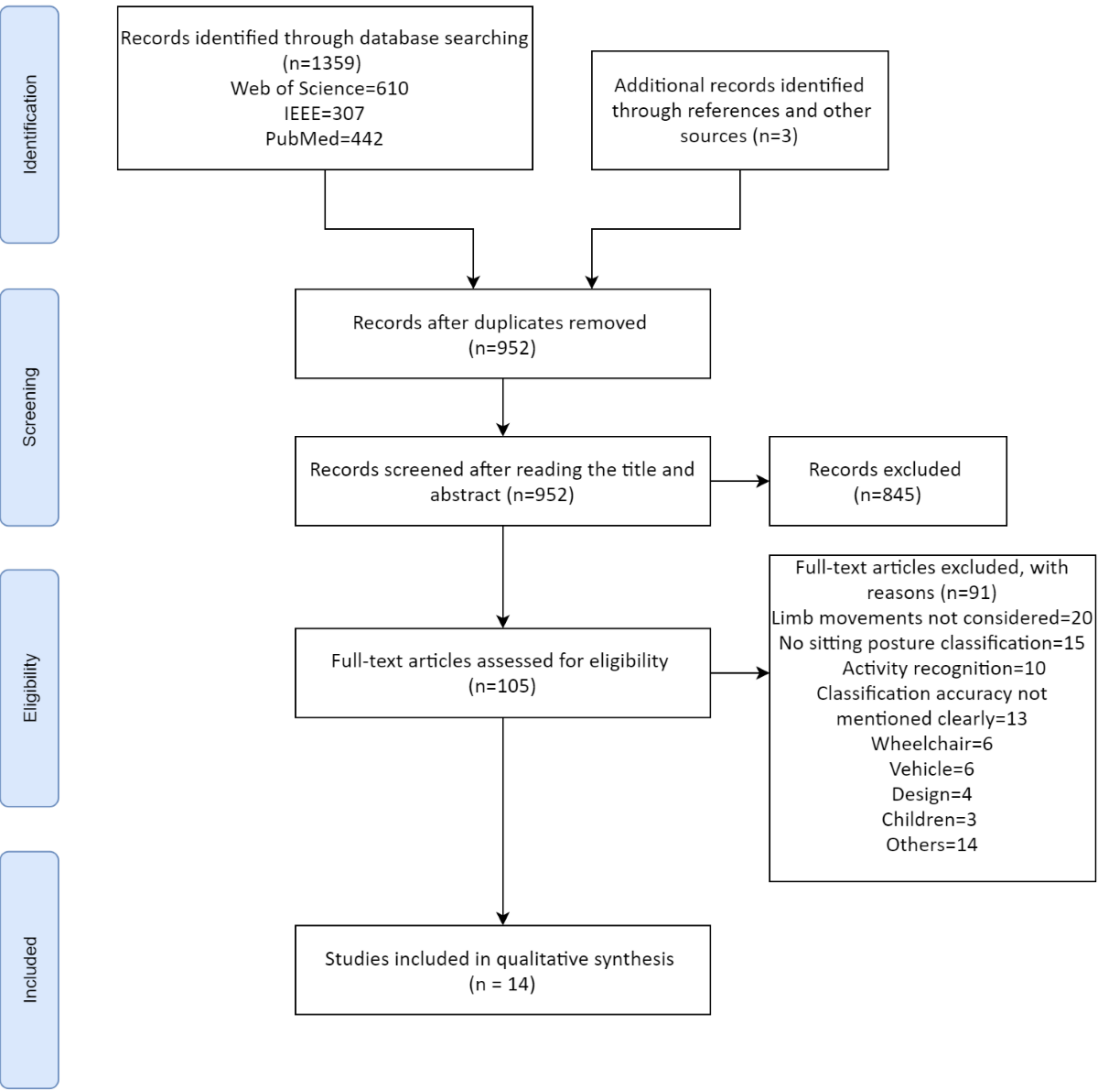
The literature search strategy using the PRISMA (Preferred Reporting Items for Systematic Reviews and Meta-Analyses) flowchart.

The shortlisting of the papers was based on the PRISMA (Preferred Reporting Items for Systematic Reviews and Meta-Analyses) flowchart presented in Figure 2 [31]. The search terms given in Table 1 were used with minor modifications to identify and retrieve 1359 potentially relevant papers: 610 from Web of Science, 307 from IEEE Xplore, and 442 from PubMed. The elimination of duplicates from these retrieved papers resulted in 949 papers. A total of 3 additional papers were included from the reference search and other sources. After screening them by reading the titles and abstracts, 105 papers were shortlisted. The excluded papers were related to air embolism, human activity recognition, gait analysis, hypertension, and other topics unrelated to sitting postures. Only 14 of the 105 shortlisted papers were selected after reading the complete papers, based on the inclusion criteria. The reasons for excluding the remaining 91 papers are provided in Figure 2. In total, 5 of the selected papers are from journal publications. A summary of the shortlisted papers is presented in Tables 4 and 5.

**Table 4:** Summary of the reviewed papers.

Study	Number and type of postures	Classification accuracy
Ma et al [30]	5 types: US^a^, LF^b^, LB^c^, LR^d^, and sitting cross-legged	SVM^e^: 95.33% accuracy; K-means clustering: 89.35% accuracy
Zemp et al [14]	7 types: US, LF, LB, LL^f^, LR, the left leg crossed over the right, and the right leg crossed over the left	Multimodal regression: 90.4% accuracy; NN^g^: 90.4% accuracy; RF^h^: 90.9% accuracy; combination of boosting, NN, and RF: 90.8% accuracy
Xu et al [13]	7 types: US, LF, LB, LL, LR, right foot over left, and left foot over right	Dynamic time warping: 85.9% accuracy
Martins et al [15]	Experiment A: 11 types: US, LF, LB, LL, LR, LB with no lumbar support, the right leg crossed, the right leg crossed with LL, the left leg crossed, the left leg crossed with LR, and slouching; experiment B: 8 types: US, LF, LB, LL, LR, LB with no lumbar support, the right leg crossed, and the left leg crossed	Experiment A: artificial NN: 70% accuracy; experiment B: thresholding and artificial NN: 93.4% accuracy
Zemp et al [16]	7 types: US, LF, LB, LL, LR, crossed legs right over left, and crossed legs left over right	RF: 82.7% accuracy
Kamiya et al [17]	9 types: US, LF, LB, LL, LR, the right leg crossed, LR with the right leg crossed, the left leg crossed, and LL with the left leg crossed	SVM: 98.9% accuracy known subject; SVM: 93.9% accuracy unknown subject
Liu et al [18]	8 types: US, LF, LB, LL, LR, crossed legs right over left, crossed legs left over right and slouching	Convolutional NN: 98% accuracy; back propagation NN: 92.8% accuracy
Pereira et al [19]	12 types: US, LF, LB, LL, LR, LB with no lumbar support, the right leg crossed, the right leg crossed with LL, the left leg crossed, the left leg crossed with LR, left leg over right, and the right leg over left	Artificial NN: 80.9% accuracy
Zhu et al [20]	10 types: US, LF, LB, LL, LR, the right leg crossed, the left leg crossed, LL with the right leg crossed, LR with the left leg crossed, and slouching	k-nearest neighbor: 81% accuracy; principal component analysis: 86% accuracy; linear discriminant analysis: 81% accuracy; sliced inverse regression: 86% accuracy; NN: 80% accuracy
Bontrup et al [1]	7 types: US, LF, LB, LL, LR, crossed legs right over left, and crossed legs left over right	RF: 90% accuracy
Mutlu et al [21]	10 types: US, LF, LB, LL, LR, the left leg crossed with LR, the right leg crossed, slouching, the left leg crossed, the right leg crossed with LL	Tekscan: 31 sensor SimpleLogistic: 87% accuracy; Prototype Sensor System: 19 sensors SimpleLogistic: 78% accuracy
Huang et al [22]	8 types: US, LF, LB, LL, LR, slumped sitting, the right leg crossed, and the left leg crossed	Artificial NN: 92.2% accuracy
Wang et al [23]	6 types: US, LF, LB, the left leg crossed, the right leg crossed, and astride sitting	Decision tree: 99% accuracy
Noh et al [24]	9 types: US, LF, LB, LL, LR, left leg trembling, right leg trembling, left leg twisted, and the right leg twisted	Triangle center: 98% accuracy

^a^US: upright sitting.

^b^LF: lean forward.

^c^LB: lean backward.

^d^LR: lean right.

^e^SVM: support vector machine.

^f^LL: lean left.

^g^NN: neural network.

^h^RF: random forest.

**Table 5:** Summary of the reviewed papers.

Study	Population	Duration of the study	Number and type of sensor(s) and location
Ma et al [30]	6 subjects	Each posture was held for 5 min, but data were collected after 1 to 2 min.	One triaxial accelerometer and cervical spine
Zemp et al [14]	41 subjects	Each posture was held for 5 s.	17 pressure sensors: 10 pressure sensors were fixed within the seat pan, 4 were fixed on the backrest, 3 were fixed on each armrest, and 1 accelerometer sensor at the rear of the backrest
Xu et al [13]	25 subjects	—^a^	256 pressure sensors in a cushion placed on the seat of the chair
Martins et al [15]	Experiment A: 30 subjects; experiment B: 30 subjects	Experiment A: each subject held each posture for 20 s; experiment B: each subject held each posture for 15 s.	8 pressure sensors or cells: 4 in the seat pad and 4 in the backrest
Zemp et al [16]	20 subjects	Free-living environment recording was for 330 min.	64 pressure sensors mat placed on the seat pan
Kamiya et al [17]	10 subjects	Each posture was maintained for 2 to 3 s.	64 pressure sensors sheet placed on the seat of the chair
Liu et al [18]	25 subjects	—	1024 pressure sensors array placed on the chair
Pereira et al [19]	72 subjects	Each subject had each posture for 20 s.	8 pressure sensor (air bladder): 4 in the seat pad and 4 in the backrest
Zhu et al [20]	50 subjects	—	Two 2016 pressure sensor sheets mounted on the seat pan and the backrest of the chair
Bontrup et al [1]	64 call center employees	Data were collected from each participant for almost 6.2 (SD 1.5) h	196 pressure sensors mat fixed to the seat pan of an office chair
Mutlu et al [21]	Tekscan: 52 subjects; Prototype Sensor System: 20 subjects	—	Tekscan: 2016 pressure sensor mat each placed on the backrest and the seat; Prototype Sensor System: 19 pressure sensors optimally placed on the backrest and on the seat of the chair.
Huang et al [22]	—	Each posture maintained for 5 s.	2288 pressure sensor (a piezoresistive sensor) on the seat
Wang et al [23]	5 subjects	Data were collected with each posture for 30 s, followed by 10 s rest.	8 pressure sensor (capacitive proximity sensor): 4 sensors on the seat and 4 sensors on the backrest
Noh et al [24]	10 subjects	Data were collected for 10 min.	8 pressure sensors on the seating area

^a^Data not available.

### Study Quality Assessment

The quality assessment of papers was performed based on the evaluations of the questionnaires in Table 2. All papers had a low bias in their aims (Q1), in their reliability in reporting the accuracy of the algorithms (Q11), and when stating their findings (Q3). Most papers were rated low regarding the justification of the number of subjects enrolled (Q6) and the eligibility criteria (Q8) used to recruit them into the studies. Other factors for the lower rating were that most papers did not mention ethics approval and written informed consent from subjects (Q9), the justification of sensor positioning (Q10), the use of cross-validation to evaluate the algorithms (Q12), and the study’s limitations (Q4). Due to the aforementioned factors, most papers were rated as medium quality, and only 1 study [14] was as rated high quality. Therefore, for all the included papers’ total median assessment score was rated as 13.5 (on a scale of 0 to 26).

### Technology

This section describes and investigates the types of sensors used, their quantity, and their placement. All studies, except for 1 study out of the 14 shortlisted studies [30], have used pressure sensors to classify sitting postures. These pressure sensors were used in the form of smart cushions, pressure mats, pressure sensor sheets, or as individual pressure sensors. In the experiment conducted by Zemp et al [14], an additional triaxial accelerometer sensor was used that was placed in the backrest of the seat to access the global chair movement and angle of the backrest. The experiment conducted by Ma et al [30] is the only study in which a triaxial accelerometer sensor was used to measure the sitting posture. An accelerometer was placed on the cervical spine to study the seated posture.

In previous studies [1,13,16-18,20-22], pressure sensors were used in the form of an array of sensors and placed on the chair’s seat pan. In 2 papers, the sensor arrays were placed on the backrest of the chair [20,21]. The sizes of these sensor arrays varied between 64 and 2288 sensors.

In some papers [14,15,19,21,23,24], sensors were sparsely placed, varying from 7 to 17. In previous studies [15,19,23], the sensors were placed such that the ischial tuberosity, the thigh region, the lumbar region of the spine, and the scapula had better contact with the sensors. The variations in the pressure distributions in these regions were distinct for different postures. In 1 of the papers, pressure sensors were additionally placed in the armrest [14]. In the study by Mutlu et al [21], ideal positions for the sensors were identified using approximation algorithms. These placements were based on the classes and features extracted from the pressure sensors. A total of 7 studies used commercial sensors in their experiments [1,14-16,20,21,24]. These commercial pressure sensors were either from Tekscan, Interlink, Sensomative, or Honeywell. The rest of the authors had designed custom-made pressure sensors.

### Study Design

This section describes the environment of the study, the duration of the study’s recording, and the number of subjects recruited. Most of the studies were conducted in a controlled environment, and the subjects were asked to follow the protocol designed for that study. In these studies, the recording duration for each posture was between 5 seconds to 3 minutes. A total of 4 papers did not mention the duration of the study [13,18,20,21]. Two studies [1,16] were designed in an occupational (free-living) setting with a sitting duration from 3 hours to 6.2 hours. In these 2 studies, the subjects were free to choose their postures as they worked. All studies in this paper had recruitment numbers varying between 6 and 72 subjects. Except for 1 study [22], all mentioned the number of subjects.

### Classification Algorithms

Different algorithms applied to differentiate and identify the various sitting postures, the type of features, and the number of postures classified are investigated in this section. NNs with varying parameters of neurons, layers, transfer function, and backpropagation methods were used for classifying the sitting postures in the majority of the studies [14,15,18-20,22]. Support vector machines [17,21,30] and random forest (RF) [1,14,16] were the second most used models. Furthermore, algorithms such as K-means [30], multimodal regression [14], boosting [14], dynamic time warping [13], k-nearest neighbors [20], sliced inverse regression (SIR) [20], decision tree [23], Naive Bayes [21], SimpleLogistic [21], linear discriminant analysis [20], principal component analysis (PCA) [20], and triangle center [24] were deployed in the papers to classify sitting postures. In this study, 6 papers [14,15,18,20,21,30] compared the performance of classification algorithms using more than one classifier. Please refer to Table 4 for more information on the classification accuracy.

Training of the classification algorithm and its accuracy depends on the type of features used and its sample size. Different types of features have been used in papers to train and test classification algorithms. Ma et al [30] extracted features from the accelerometer using PCA. Zemp et al [1,14,16] used the median of the sensor data’s 1-second duration as the features. In the study by Xu et al [13], the two-dimensional pressure data were converted into one-dimensional data, and the similarity between the signals was used as the feature. In 2 papers [15,19], data collected for each posture from the pressure sensors were divided into groups, and the average of each group was used as a feature. Mutlu et al [21] obtained the position and size of the bounding box; distance of the bounding boxes; the distance and angle between the centers of the pressure areas of the seat and backrest; the centers, radii, and orientations of 2 ellipses from the seat; and the pressure applied to the bottom area as the features to train the algorithm. Huang et al [22] used 40 frames of collected pressure data for each position as the training data. Wang et al [23] used the average and SD of the pressure of the sensors as features. Noh et al [24] used the distance between the center points, intensity, and frequency size of the pressure sensor movements between the current frame center and the previous to train the algorithm.

In the reviewed papers, the number of sitting postures classified varied between 5 and 12. The most common postures observed in Table 4 were upright sitting lean forward, lean backward, lean right, and lean left, as shown in Figure 1. The rest of the postures mentioned in the papers are slight variants of these postures and include different limb movements. Noh et al [24] has also considered in their studies trembling and twisting of the right and left legs.

### Algorithm Performance

The evaluation of the algorithms must understand the accuracy, sensitivity, and positive predictive value and check if there is overfitting of the algorithm. Most classification algorithms use confusion matrices to evaluate the posture’s classification accuracy. The confusion matrix gives the accuracy of the algorithm and helps interpret the data and the posture that has been misclassified as some other posture. The confusion matrix helps analyze misclassification and rectify errors using each posture’s sensitivity and positive predictive value. Of the 14 papers [1,13,15-17,19,21-24], 10 used a confusion matrix to evaluate the performance of the classification algorithm.

Overfitting is another challenge when training an algorithm for machine learning. This occurs when the algorithm fits the training data set and not on a new data set. To check that the algorithm is not overfitting, 6 papers [1,14-17,21] used either 10-fold or leave-one-out cross-validation (internal validation). External validation was performed in the study by Liu et al [18] using 5 external test data sets instead of just cross-validation. Thus, the evaluation and overfitting of the algorithms need to be checked for each classification algorithm.

## Discussion

### Principal Findings

The WHO and other authors [1,3,9] emphasize the monitoring of sitting postures in free-living environments as a way to understand the mechanical factors involved in musculoskeletal discomfort and pain such as LBP. Different types of sensors (eg, pressure and triaxial accelerometer sensors) and algorithms could be used to accomplish this task. Therefore, this study intends to reveal the current state-of-the-art and the involving algorithms and sensors to classify sitting postures. In each of the included studies, we investigated the type of sensors, the algorithms used, the number of postures classified, the study design, and the environment in which these studies were conducted.

### Study Quality Assessment

In summary, most of them had an overall medium quality, with a median score of 13.5. Most of the included papers were conference papers, and only 5 papers were published as journal papers. Among the 5 journal papers, we rated only 1 as high quality. Most of these papers neither had a straightforward study design nor were their results cross-validated. Thus, the sitting posture classification research is still preliminary and requires further investigation to evaluate the findings. Therefore, to evaluate the findings more systematically in future studies, we recommend that the study be designed carefully. The classification results need to be cross-evaluated such that newer studies can replicate the findings. 

### Technology

The majority of the papers in this study reported pressure sensors positioned on a chair to classify sitting postures. In these studies, the authors could distinguish between postures because of changes in pressure intensity at different body locations. Arrays of such pressure sensors were used in the backrest and seat [1,13,16-18,20-22] in these studies. However, this is an inefficient use of sensors. As we know from the other studies [15,19,23], the sensors can be strategically placed on the chair in direct contact with the ischial tuberosity, thigh, lumbar, and scapular regions of the subject’s body. Such positioning can show the changes in the pressure intensity by using fewer sensors. Nevertheless, while using pressure sensors, researchers must ensure that the subjects empty their pant pockets, as this could hamper the results by changing the pressure intensity further [15,19]. Furthermore, the study conducted by Mutlu et al [21] concluded that the number of pressure sensors should be decreased to reduce the cost of hardware and improve the classification accuracy. Therefore, to improve the classification accuracy and reduce the hardware cost, we suggest the strategic placement of sensors. Thus, the type of sensor and its location must be carefully considered while performing the study.

Regarding the classification of postures, as the number of distinct postures increased, the accuracy of the algorithm decreased [1,16], as the differentiation between the postures was challenging. Therefore, in some studies that used pressure sensors, specific postures were merged into a single posture, resulting in the loss of similar postures. One possible solution to overcome this challenge is to measure the spine movement. Spine movements can be used to differentiate between similar but distinct postures. In the study conducted by Ma et al [30], a triaxial accelerometer was placed at a random location on the cervical spine to measure spine movements to classify sitting postures. However, this was not enough to measure the spine movement, as the upper and lower parts of the spine are independent in motion [32,33]. Accordingly, we propose using multiple sensors, which measure the orientation (eg, inertial measurement unit [IMU] sensors) of the upper and lower spine to be employed to differentiate and classify similar postures. IMUs used in movement trackers in clothing is another option that could be considered if the sensors are placed at the right location on the spine.

### Study Design

Most studies were performed in a controlled environment, with subjects being asked to sit in specific postures. However, in free-living conditions, the number of postures could vary and might not match those performed in controlled environments. Moreover, sitting postures depend on the subject’s spinal curvature, intradiscal pressure, tissue stress, and muscle activation [34,35]; however, most studies did not investigate these factors in depth. Therefore, we strongly recommend that communities perform sitting posture classification in free-living environments. The sitting posture classification is personalized and can be translated into real-life sitting.

### Classification Algorithms

The accuracy of the classification algorithms depends on the type of features, location of the sensors, number of subjects, sample size of the data used for training the algorithm, and number of postures classified. For example, the studies that had the highest number of subjects (72, 64, 52, and 50) had maximum accuracies of 80.9% (Artificial NN), 90% (RF), 87% (SimpleLogistic), and 86% (SIR), respectively [1,19-21]. On the basis of the comparison of the number of classes and the duration of the experiment, the RF algorithm appears to be suitable for classification using pressure sensors. However, the conclusion that the RF algorithm performs well could be biased; it is still too early to conclude that the RF algorithm has the best performance, as there were only 7 postures involved; and the information regarding evaluation using labels was not provided. On the basis of these findings, we advise that the assessment of the predictions of these algorithms should not be based only on the overall accuracy of the system but also on the classification accuracy of each posture and the sample size.

After analyzing the *Type of postures* column in Table 4, we can infer that there are 5 main sitting postures: upright sitting, lean forward, lean backward, lean left, and lean right. Other similar postures are the combination of these postures along with spine and limb movements. Limb movements are an essential aspect of understanding musculoskeletal discomfort and pain [25,27]. Studies have revealed that cross-legged sitting results in asymmetries in the spine and pelvic shapes and increases external oblique muscle activities [26]. Therefore, limb movements must be considered while performing the classification. Furthermore, the postures can be subclassified based on the spine movement. For example, there are 2 types of sitting postures in upright sitting: thoracic upright sitting and lumbopelvic upright sitting. Therefore, in the subclassification, the spine curvature should also be identified along with the posture type, as the curves provide insight into the type of strain the lower back undergoes [36]. Thus, we would point out that future researchers perform subclassification of postures based on spine and limb movements and 5 basic sitting postures.

Static sitting postures are associated with musculoskeletal discomfort and pain. Hence, it is important to understand how frequently a subject moves while monitoring sitting postures [1,16]. Therefore, static sitting postures must be differentiated from dynamic sitting postures. Hence, 2 studies measured transitional periods [1,16], representing the change of one posture to other. Transitional periods also indicate whether a person with static sitting posture has made changes. Therefore, transitional periods must be considered when classifying sitting postures. However, the types of static postures that indicated the presence of musculoskeletal discomfort and pain were not mentioned. Therefore, to understand the cause of musculoskeletal discomfort and pain, we urge future studies to unambiguously classify the type of static sitting posture while considering the transitional periods for differentiating between static and dynamic postures.

### Algorithm Performance

In the shortlisted papers, there was no mention of the use of labels for evaluating the accuracy of the classification. An exclusive annotation of labels is not required in studies using a defined protocol. However, when the study is performed in a free-living environment, exclusive annotation of labels is needed to evaluate the performance of the classification algorithm. One solution for labels is the self-reporting of sitting postures by the subject. However, this was limited by the duration of the measurements. Over a longer time, the subjects may become unaware of many movements while concentrating on their work. The challenge of self-reporting of labeled postures can be overcome using video cameras [37]. In the future, we will use a three-dimensional camera as a ground truth that can be used to verify the predicted activity at any particular time instant.

During the evaluation, it is also important to check if there is an overfitting of the algorithm. In this study, most of the included studies prevented overfitting by performing cross-validation or by using a new data set for testing. In one of the studies, when the algorithm’s training and testing were performed on a data set collected from a known subject, the classification accuracy was 98.9% after cross-validation. However, when training and testing were performed with 10-fold cross-validation using 9 subjects’ data for training and 1 subject’s data for testing, the accuracy decreased to 93.9% [17]. Therefore, it is crucial to evaluate the robustness of an algorithm even when the same subject is not used to train the algorithm.

### Conclusions

This study has been conducted to understand the types of sitting postures in the context of spine and leg movements, as sitting for long hours is related to musculoskeletal discomfort and pain.

The quality appraisal shows that future studies need to provide a more precise description of the study design and validation to replicate the studies. The following 5 main sitting postures were present in most of the studies evaluated: upright sitting, lean forward, lean backward, lean left, lean right, and different combinations of limb and spine movements. However, a deeper understanding of spine orientation and variations in those sitting postures are still needed for a more personalized assessment in the context of musculoskeletal discomfort and pain. This is because the individual posture relies on spine curvature, that is, even upright sitting differs from person to person. Even the same person does not always maintain the same posture as the postures instructed in the laboratory. Therefore, it is essential to perform these studies in a free-living environment to understand people’s actual postures and reduce the bias from experiments in a controlled environment. To accomplish these studies in a free-living environment in the future, we recommend using multiple sensors that can measure three-dimensional spine movement and angle, such as IMUs. Furthermore, we suggest using labels to evaluate the classification of sitting postures and cross-validation of the algorithms to avoid overfitting to a specific data set. Three-dimensional cameras could be recommended for initial studies to obtain labels. Finally, we recommend the measurement of transitional periods to shed light on more factors affecting musculoskeletal discomfort and pain.

### Limitations

In this study, we only included papers written in English, excluding papers written in other languages. Furthermore, with the strict exclusion criteria, there is a possibility that this study missed some additional methods for sitting posture classification. Moreover, we did not include a quantitative analysis (eg, meta-analysis) because of the high heterogeneity in subject characteristics, experimental design, and algorithms. Finally, systematic technological reviews would benefit from standardized methods to assess the risk of bias and systematic content creation, similar to the PRISMA guidelines used in medical and life sciences. Therefore, we believe that the research community must invest in more standardized systematic reviews in such interdisciplinary areas.

## Appendix

Multimedia Appendix 1 The search terms for the three database search engines.

## Abbreviations

IMU: inertial measurement unit
LBP: low back pain
NN: neural network
PCA: principal component analysis
PICO: Population or Problem, Intervention or Exposure, Comparison, and Outcome
PRISMA: Preferred Reporting Items for Systematic Reviews and Meta-Analyses
RF: random forest
SIR: sliced inverse regression
WHO: World Health Organization
