# Supplementary material for: Monitoring of Sitting Postures With Sensor Networks in Controlled and Free-living Environments: Systematic Review
Source: JMIR Biomed Eng. 2021 Mar 1;6(1):e21105. doi: 10.2196/21105 (PMC11041431; doi:10.2196/21105)
Supplement: Multimedia Appendix 1 [file biomedeng_v6i1e21105_app1.pdf]

Appendix A

The search terms for the three database search engines are as follows:

PubMed:

((((((sitting OR seating OR seated) AND (posture* OR position OR behaviour)) AND (sensor+ OR "inertial measurement unit" OR "IMU" OR wearable OR pressure OR piezoresistive OR accelerometer OR gyroscope) AND (algorithm*[Mesh] OR *supervised OR classif* OR detection OR OR recognition))) NOT Animals))

Web of Science:
TS=((((((((sitting OR seating OR seated) AND (posture* OR position OR behaviour)) AND (sensor+ OR "inertial measurement unit" OR IMU OR wearable OR pressure OR piezoresistive OR accelerometer OR gyroscope) AND ("machine learning" OR "neural network*" OR algorithm* OR *supervised OR classif* OR detection OR identification OR recognition)))))) NOT (Animals))

IEEE Xplore:
(((((((sitting OR seating OR seated) AND (posture* OR position OR behaviour)) AND (sensor+ OR "inertial measurement unit" OR IMU OR wearable OR pressure OR piezoresistive OR accelerometer OR gyroscope) AND ("machine learning" OR "neural network*" OR algorithm* OR *supervised OR classif* OR detection OR identification OR recognition OR "artificial intelligence"))) NOT Animals)))
